# Repeated sessions of bilateral transcranial direct current stimulation on intractable tinnitus: a study protocol for a double-blind randomized controlled trial

**DOI:** 10.12688/f1000research.13558.1

**Published:** 2018-03-14

**Authors:** Arash Bayat, Miguel Mayo, Samaneh Rashidi, Nader Saki, Ali Yadollahpour

**Affiliations:** 1 Hearing Research Center, Ahvaz Jundishapur University of Medical Sciences, Ahvaz, 61357-15794, Iran; 2 Department of Otorhinolaryngology, A Coruña University Hospital Complex, A Coruña, 15006, Spain; 3 Bioelectromagnetic Clinic, Imam Khomeini Hospital, Ahvaz Jundishapur University of Medical Sciences, Ahvaz, 61936-73111, Iran; 4 Department of Medical Physics, School of Medicine, Ahvaz Jundishapur University of Medical Sciences, Ahvaz, 61357-33118, Iran

**Keywords:** Transcranial direct current stimulation, Repeated sessions, bilateral, Intractable chronic tinnitus, Tolerability, adverse effects

## Abstract

**Background**: Transcranial Direct Current Stimulation (tDCS) is reportedly a potential treatment option for chronic tinnitus. The main drawbacks of previous studies are short term follow up and focusing on the efficacy of single session tDCS. This study aims to investigate the therapeutic efficacy, adverse effects (AEs) and tolerability of repeated sessions of bilateral tDCS over auditory cortex (AC) on tinnitus symptoms

**Methods**: This will be a double-blinded randomized placebo controlled parallel trial on patients (n=90) with intractable chronic tinnitus (> 2 years) randomly divided into three groups of anodal, cathodal, and sham tDCS. In the sham treatment, after 30 sec the device will be turned OFF without informing the patients. The tDCS protocol consists of 10 sessions (daily  20 min session; 2 mA current for 5 consecutive days per week and 2 consecutive weeks) applied through 35 cm ^2^ electrodes. The primary outcome is tinnitus handicap inventory (THI) which will be assessed pre- and post-intervention and at one month follow-up. The secondary outcomes are tinnitus loudness and distress to be assessed using a visual analogue scale (VAS) pre-intervention, and immediately, one hour, one week, and one month after last stimulation. The AEs and tolerability of patients will be evaluated after each session using a customized questionnaire. Possible interactions between the disease features and treatment response will be evaluated.

**Discussion**: To our knowledge this is the first study to investigate the effects of repeated sessions of tDCS on chronic tinnitus symptoms with one month follow-up. In addition, the AEs, and tolerability of patients will be studied. In addition, the possible interactions between the disease specific features including the hearing loss, laterality, type of tinnitus, and treatment response will be evaluated.

**Trial registration**: The study has been registered as a clinical trial in Iranian Registry of Clinical Trial ( IRCT2016110124635N6) on the 01/06/2017.

## Abbreviations

tDCS: transcranial direct current stimulation; AC: auditory cortex; DLPFC: dorsolateral prefrontal cortex; THI: tinnitus handicap inventory; BDI-II: Beck Depression inventory; BAI: Beck Anxiety Inventory; dBHL: decibels hearing level.

## Background

Tinnitus is a subjective auditory phantom perception without an external physical sound source ^1^, which exists in different forms including pulsatile or continuous, buzzing, ringing, hissing, tone, or a combination of them ^2^. It is a relatively common disorder with 10–15% prevalence among adults worldwide. Tinnitus is a debilitating condition which severely affects the quality of life. It is accompanied by different comorbidities such as anxiety, depression, and sleep disturbances ^3^.

 There is no definitive treatment for tinnitus and a large proportion of tinnitus become intractable ^1, 3^. During the recent years, different non-pharmacological techniques have been developed for treatment of chronic tinnitus including cognitive behavioral therapies, hearing aids, neurofeedback, and noise-masking techniques ^4– 6^. Although some of these techniques have shown therapeutic outcomes, the therapeutic efficacies of these techniques are limited and studies to develop more efficient techniques are ongoing.

Transcranial direct current stimulation (tDCS) is a non-invasive neuromodulation technique and recent studies have reported promising therapeutic outcomes in improving cognitive functions in healthy individuals ^7^ as well as symptoms of different neuropsychiatric disorders such as depression ^8^, obsessive compulsive disorder ^9^ auditory-verbal hallucinations ^10^, and chronic pain ^11^. Several studies have investigated the therapeutic efficacy of different tDCS protocols in chronic tinnitus ^11– 15^. Results were in general promising, despite some controversial findings. In treating tinnitus with tDCS, there are two main targets as the site of stimulation including the dorsolateral prefrontal cortex (DLPFC) ^16, 17^, and the auditory cortex ^18– 20^. The neural anomalies, hyperactivity, or maladaptive plasticity of the primary and secondary auditory cortices have been reportedly the main causes of tinnitus loudness and disturbances in non-auditory structures and networks such as anterior cingulate cortex, and particularly DLPFC are the main causes of distress or annoyance experienced in tinnitus ^2, 3, 21^. The main hypothesis for therapeutic application of tDCS in tinnitus is disturbing ongoing abnormal neural activities responsible for tinnitus. According to this approach, the areas involved in the pathophysiology of tinnitus are targeted by tDCS. The other hypothesis of eliciting therapeutic outcomes from tDCS is inducing plastic changes to alter the maladaptive plasticity of the involved regions. The important point for treatment of tinnitus is that there is a large network consisting of different auditory and non-auditory regions with overlapping functions ^22– 24^.

Most of the studies performed to date, have focused on the effects of single tDCS session on tinnitus symptoms, with short term follow ups ranging some hours to some days. To our knowledge, there is no published study investigating the effect of repeated sessions of tDCS over the auditory cortex (AC) on tinnitus symptoms as well as the AEs of and tolerability to the tDCS with one month follow up. This study aims to investigate the effects of repeated sessions of tDCS on tinnitus symptoms with one month follow-up. Our main hypothesis is that repeated sessions will lead to aggregative therapeutic effects on tinnitus. In addition, we want to evaluate possible association between treatment response, and patient's and tinnitus specific characteristics including gender, THI basal score, laterality, tinnitus quality, duration, hearing loss class, etc.

## Methods/design

### Participants

This is a part of a larger project which is designed to comprehensively investigate the efficacy of different tDCS protocols at different sites of brain for treatment of intractable chronic tinnitus. In this double blinded placebo controlled randomized trial will be conducted on intractable tinnitus patients (n=90) who will be randomly divided into three groups of anodal (anode/cathode on left/right AC), cathodal (cathode/anode on left/right AC, and sham (anode/cathode on left/right AC) tDCS. In the sham treatment, after 30 sec the device will be turned off without informing the patients. The experimental procedures of the study including tDCS interventions and the evaluations of the outcomes will be performed in the Bioelectromagnetic Clinic in Ahvaz Imam Hospital, Ahvaz, Iran. Patients with intractable chronic tinnitus (> 2 years) will be enrolled in this study. This is a single-center, non-stratified, with balanced randomization [1:1:1], double-blind, placebo-controlled, parallel-group study conducted in Bioelectromagnetic Clinic, Imam Khomeini Hospital, Ahvaz, Iran. Allocation concealment will be performed by random allocation cards using computer-generated random numbers. The patients will be randomly assigned into three groups of cathodal, anodal, or placebo tDCS.

### Ethics approval and consent to participate

All of the experimental procedures of this study were approved by the local ethics committee of Ahvaz Jundishapur University of Medical Sciences, Ahvaz, Iran (registration code: IR.AJUMS.REC.1394.639) which were in complete accordance with the ethical standards and regulations of human studies of the Helsinki declaration (2014) ^25^. After enrolment and before the start of the study, researcher will clearly explain the experimental procedures, the objectives, possible benefits, and side effects of the study to the patients and then all participants will fill and sign a written consent form for contribution in the study. The study was registered as a clinical trial in the Iranian registry of clinical trials ( IRCT2016110124635N6).

### Inclusion and exclusion criteria

The patients should meet specific criteria for inclusion in this project. The primary criteria will be evaluated during the neurological and otolaryngological examinations by experienced specialists. The patients will be selected among the tinnitus patients referred to the Tinnitus Clinic in the Khuzestan Cochlear Implant Center, Ahvaz, Iran. After the start of the study and anytime during the study, patients have the right to leave the study. In case of any significant adverse effects (AEs), the procedures for the respective patients will be terminated.

**Inclusion criteria**

• Idiopathic chronic and medication-resistant tinnitus (more than 2 years)
• Age range of 18 to 70 years old
• No use of medications at the time of intervention

**Exclusion criteria**

• History of epileptic seizures, brain trauma
• Severe psychotic and psychiatric disorders
• Concurrent severe vertigo
• Meniere's disease
• Severe organic comorbidity
• Using pacemaker or defibrillator
• Present pregnancy, neurologic disorders such as brain tumors,
• Concurrent treatment for mental disorders.

All prospective subjects will undergo complete audiometric and neurologic examinations by experienced specialists. The experimental procedures of the study including tDCS interventions and the evaluations of the outcomes will be performed in the Bioelectromagnetic Clinic in Ahvaz Imam Hospital, Ahvaz, Iran.

### Study protocol

This study is a double-blind randomized controlled clinical trial design and patients will be randomly assigned into three groups of anodal and cathodal tDCS and sham tDCS. The random allocation will be performed based on balanced randomization [1:1:1] where the allocation concealment will be applied by random allocation cards using computer-generated random numbers. The three groups will be matched for age, gender, ethnicity, and audiometric main characteristics. To reduce the procedure and subjective bias, the patients, the researchers who will evaluate the outcomes, and the researchers who perform data analyses will be blinded on the type of protocol. In addition, the blinding quality of the study will be assessed after completion of the intervention.

### Transcranial direct current stimulation protocol

The direct current will be applied through a saline-soaked pair of surface electrodes (35 cm ^2^) and delivered by a tDCS device which is a specially developed, battery-driven, constant current stimulator with a maximum output of 2 mA. The tDCS device used in this study will be an OASIS Pro ^TM^ device by Mind Alive Inc (Edmonton, Alberta, Canada). Anodal and cathodal tDCS groups will consist of daily 20-min sessions of 2 mA current for five consecutive days per week and for 2 consecutive weeks (10 sessions in total). In anodal tDCS, the anode and cathode will be respectively centered over left AC (halfway T3 - F7) and right AC (halfway T4 - F8). In the cathodal tDCS the electrodes position will be reversed where cathode and anode will be respectively centered over left AC (halfway T3-F7) and right AC (halfway T4-F8). The site of electrodes will be selected according to the International 10-20 electroencephalography system. In the sham tDCS group, the electrode montage will be the same as the anodal tDCS, but the device will be turned off after 30 s without the knowledge of the participant. These parameters for sham stimulation were chosen based on previous reported findings where the perceived sensations of stimulation on the skin, such as tingling, usually disappear in the first 30 s of active tDCS ^23, 26^ ( Table 1).

**Table 1.** Setting for anodal, cathodal, and sham Transcranial Direct Current Stimulation (tDCS).

	Anodal tDCS	Cathodal tDCS	Sham tDCS
Current density	57.1 µA/cm ^2^	57.1 µA/cm ^2^	57.1 µA/cm ^2^
Electrode montage	Anode left (halfway T3 - F7) and cathode right AC (halfway T4 - F8)	Cathode left (halfway T3 - F7) and anode right AC (halfway T4 - F8)	Anode left (halfway T3 - F7) and cathode right AC (halfway T4 - F8)
Duration	20 min	20 min	20 min ^*^

Note: * 30 seconds after the start of the stimulation the device will be turned off without the knowledge of patient.

### Adverse effects and tolerability

The previous studies on the safety and tolerability of tDCS have shown that the 2 mA current applied in 20 minutes are associated with no serious AEs ^24, 26^. The common reported AEs are itching or tingling sensation, mild headache or fatigue and local burns at the site of stimulation. However, most of the AEs and tolerability assessments have been performed on the tDCS were for single session ranging 20–30 minutes and current of 1 to 2 mA. In our study, we will use repeated sessions of tDCS (2 mA current for 20 minute, total 10 sessions, daily one session, five consecutive days per week and 2 consecutive weeks), thus the reported AEs in the previous studies may be intensified. Therefore, we will assess the AEs using a customized questionnaire ( Supplementary File 1). The questionnaire will be performed during and after each tDCS session to record all the perceived AEs from the patients in all three tDCS groups.

## Outcome assessments

### Baseline evaluations

There will be different evaluations before the intervention. After enrollment, the patients will undergo complete audiometric and neurological assessments by expert specialties. The tinnitus severity for each patient will be determined through asking the patient and confirming the class by an expert otolaryngologist as buzzing, cicadas, high pitch whistling, hissing, humming, ringing, pulsating, thumping, and ticking. The tinnitus laterality and duration will be also determined.

### Hearing assessment

Hearing assessments will be performed in an acoustically isolated chamber (ISO 8253–1:2010). Pure-tone audiometry will be performed using AC 40 dual channel Audiometer (Intracoustics Co., Denmark). The hearing thresholds will be recorded over the frequency ranges of 250 to 8000 Hz for air conduction and 500 to 4000 Hz for bone conduction pathways, using the modified Hughson–Westlake Method as per recommended by ANSI 1997 ^27^. Pure-tone audiometry is considered normal when the hearing thresholds at all frequencies are below 20 decibels hearing level (dBHL). Hearing loss will be classified according to the type and degree ^28^. The class of hearing loss in both ears will be assessed as normal hearing threshold (<20 dB), mild hearing loss (20 – 40 dB), moderate hearing loss (41 – 70 dB), severe hearing loss (70 – 90 db), and profound hearing loss (> 90 db). The hearing class is determined as the average of threshold in 250 Hz, 1000, 2000, and 4000 Hz. The possible interactions of the aforementioned variables and the amount of each primary or secondary outcome will be studied.

### Outcomes assessments

The primary and secondary outcomes will be measured in two main time points: pre- and post-intervention. The pre-intervention assessments of the outcomes will be performed at the first day of the intervention before tDCS stimulation (T _1_). There will be four time points of post-intervention assessments as T _2_, T _3_, T _4_, and T _5_ corresponding to the immediately after last session, one hour, one week, and one month after last tDCS session. Table 2 represents the SPIRIT schematic protocol of the study and timing of the assessments.

**Table 2.** Standard Protocol Items: Recommendations for Interventional Trials of this study.

	Enrolment	Allocation	Pre- intervention	Intervention	Post- intervention			
TIME POINT	*-T _1_*	T _0_	*T _1_*		*T _2_*	*T _3_*	*T _4_*	*T _5_*
ENROLMENT:								
Eligibility screen	X							
Informed consent	X							
*Demographic* *information*	X							
Allocation		X						
INTERVENTIONS:								
*real (anodal and* *cathodal) tDCS*				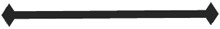				
*Sham tDCS*				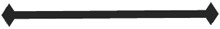				
ASSESSMENTS:								
*Neurological* *and audiological* *assessments*	X							
*Hearing loss* *assessment*	X							
*Primary outcome:* *THI*			X		X			X
*Secondary outcomes:* *loudness*			X		X	X	X	X
*Distress*			X		X	X	X	X
*AEs and Tolerability*				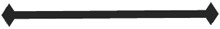	X			

Note: THI: tinnitus handicap inventory; AEs: adverse effects, -T _1_: one month before intervention, T _0_: one day before intervention, T _1_: 5 min before intervention, T _2_: immediately after last session, T _3_: at hour after last session, T _4_: one week, and T _5_: at month after last tDCS session. Real tDCS refers to anodal and cathodal tDCS groups.

***Primary outcome***. Tinnitus handicap inventory (THI) score will be the primary outcome. The THI is a 25-item questionnaire and commonly used as self-reporting inventory for disabling level of tinnitus. The THI items are grouped into three subscales: Functional (11 items), emotional (9 items), and catastrophic (5 items) subscales. Each item is three point Likert type scale with three alternative assessments: yes (4 points), sometimes (2 points), and no (0 point). The questionnaire yields scores for each subscale and a total score that ranges 0 to 100, where higher score means more severe tinnitus or a greater handicap ^29^. The THI is widely used as the primary outcome measure in double-blind trials with a control group to assess the effect of pharmacological or non-pharmacological agents in tinnitus patients. The functional subscale items reflect the effect of tinnitus on mental, social, occupational, and physical functioning. The emotional subscale items probe the individual’s emotional reactions to the tinnitus and the catastrophic response items address whether tinnitus makes the respondent feel desperate, trapped, hopeless or out of control. In this study, the THI will be assessed prior to intervention, and immediately, and at one month after the last tDCS session. Response to treatment is defined as a reduction of equal or more than 20 points in THI score (Pre-treatment THI – Post-treatment THI ≥ 20). This cut-off value for treatment response to tDCS is generally considered as significant clinical improvement in the previous similar studies ^15, 30^. 

***Secondary outcomes***

**Tinnitus loudness and distress**. The tinnitus loudness and distress will be assessed using a numeral 0–10 visual analogue scale rating scale before intervention, and immediately, one hour, one week, and one month after last tDCS session. For tinnitus loudness and distress a 10 percent or 2 point reduction of 0–10 scale will be considered as treatment response ^31, 32^.

## Blinding assessment

The previous studies have shown that the experience of the sensations induced by tDCS are usually fade out after 30 seconds; thus, we will use this period in the sham tDCS group, to evaluate the blinding quality of the study design, after completion of the intervention we will ask the patients whether they know which type of stimulation, real or sham tDCS, they received. This assessment will be performed after each tDCS session in all groups. 

***Adverse effects and tolerability***

Previous studies have reported no severe AEs of tDCS for different protocols. However, most of the previous studies used single session tDCS. For this study, considering the multisession protocol of tDCS, the AEs of and tolerability to the repeated sessions of tDCS will be assessed using a customized questionnaire ( Supplementary File 1) which will be applied after the last session.

## Statistical assessments

All statistical tests will be performed using SPSS (V20.0, IBM Corporation, New York, USA). To determine whether the anodal, cathodal, and sham tDCS groups are well-matched in terms of age, sex, quality of tinnitus, tinnitus laterality, THI score, and duration and class of hearing loss, these dependent variables will be compared between the three groups using independent samples t-tests.

For nominal data like gender and class of hearing loss, Chi-square tests will be conducted across groups. Independent sample t-tests were also used to determine if individual baseline measures differed between groups (THI, tinnitus loudness, and distress). Prior to conducting *t*-tests, Levene’s test for equality of variances was used. In no instance was Levene’s test violated; thus, equality of variances was assumed. The number of female and male participants and the quality of tinnitus symptoms, across each group will be compared using Pearson Chi-square tests. Mixed repeated-measure ANOVAs with the within‐subject factor time and the between‐subject factor stimulation were performed for evaluating changes in THI, tinnitus loudness, tinnitus distress, BDI and BAI scores. For the ANOVAs, sphericity was tested with the Mauchly’s test, and in the event of a violation of Mauchly’s test, the Greenhouse-Geisser correction was applied. In case of significant effects, follow-up post-hoc t-tests were carried out using LSD adjustments for multiple comparisons to examine if tDCS caused a significant difference relative to sham or baseline. An analysis of covariance will be performed with Time as within-subject factor, Stimulation as between-subject factor, and gender, laterality of tinnitus, duration of tinnitus as covariates. In addition, we will calculate Pearson’s correlations between change of THI and tinnitus loudness and distress values from pre- to post-stimulation.

Finally, we will examine the number of responders versus non-responders under each treatment condition (anodal, cathodal, and sham). This will be accomplished by calculating change scores between the pre-treatment and post-treatment THI values (Pre-treatment – Post-treatment). Responders will be considered individuals who showed reductions in THI score of equal or more than 20 points in the THI score (Pre-treatment THI – Post-treatment THI ≥ 20). For tinnitus loudness and distress a 10 percent or 2-point reduction of 0–10 scale is considered as treatment response. Pearson’s chi-square tests will be used to evaluate whether there are differences in the proportion of responders versus non-responders under each condition. For all statistical tests the P-value < 0.05 is defined as statistically significant level.

## Sample size calculation

The sample size of this study was calculated using the standard sample size formula (represented in Equation 1). The study of Shakhawat *et al.* was considered as the basic study to determine the sample size of our study with the predetermined power and level of significance. Considering the power of 85 percent and for confidence interval of 95 percent, the sample size of this study is 22 patients. We will use 25 patients in each group so that future attrition of the patients due to long term period of the tDCS sessions will be compromised.

$$n=\frac{{\left(z_{1–\frac{\alpha }{2}}+z_{1–\beta }\right)}^{2}(S_{1}^{2}+S_{2}^{2})}{{\left({\bar{X}}_{1}-{\bar{X}}_{2}\right)}^{2}}\;Equation\;1$$

## Discussion

Recent studies have shown beneficial effects of tDCS in improving cognitive functions in healthy individuals ^7^ and also symptoms of different neuropsychiatric disorders such as depression ^8^, obsessive compulsive disorder ^9^, auditory-verbal hallucinations ^10^, and chronic pain ^11^. The therapeutic effects of tDCS in tinnitus have been reported by several studies; however, the findings are heterogeneous and controversial. In addition, most of the conducted studies have focused on single session tDCS, with short follow-up periods ranging some hours, to a few days ^13, 16, 33^. The present study aims to investigate the therapeutic efficacies of repeated sessions of bilateral tDCS (anodal/cathodal over the left/right AC) on tinnitus symptoms, its loudness, and distress in patients with intractable chronic tinnitus. The main advantage of this study is that it is a double blinded which will remove the placebo effects of the tDCS. The other main advantage of this study is the one month follow-up which is relatively long period, compared to the previous studies. Moreover, we aim to evaluate the possible interactions between the disease specific features and treatment response to develop a more specific and disease oriented tDCS protocol. To our knowledge this will be the first study of repeated sessions of tDCS with one month follow-up. Considering the repeated sessions of tDCS in this study, we will investigate the AEs of and tolerability of the patients in this study.

## Trial status

To date, the enrollment of the patients has been performed and the allocation will be performed in the near future. The study is supposed to be started in end of January and finished in August 2018.

## Dissemination of findings

The data collection of this study is supposed to be completed August 2018 and the results of this trial will be communicated to the external funding body through a formal report. In addition, the trial results will be communicated to the public through publishing as dataset and original research in the relevant scientific journals. There is no limit in the publication of the trial results.

## Data availability

*All data underlying the results are available as part of the article and no additional source data are required*

## Supplementary materials

Supplementary File 1: The customized questionnaire of adverse effects of and tolerability to Transcranial Direct Current Stimulation (tDCS). The assessments of AEs and tolerability will be performed during and after each session.
