# Supplementary material for: Repeated sessions of bilateral transcranial direct current stimulation on intractable tinnitus: a study protocol for a double-blind randomized controlled trial
Source: F1000Res. 2018 Mar 14;7:317. [Version 1] doi: 10.12688/f1000research.13558.1 (PMC5887075; doi:10.12688/f1000research.13558.1)
Supplement: Peer review history

# Peer review: Referee response for version 1

**DOI:** 10.5256/f1000research.14726.r32356

**Published:** 2018-04-04

**Authors:** Daniel Robles-Camarillo

**Affiliations:** 1 Graduate and Research Department, Polytechnic University of Pachuca, Zempoala, Hidalgo, Mexico

1.           Summary

 This protocol aims to research the therapeutic effect of tDCS, adverse effects and tolerability of bilateral tDCS over auditory cortex on tinnitus symptoms, applied on a very specific group of patients with intractable chronic tinnitus. It is proposed an interesting a double blinded experiment with three patient groups that consider the electrodes allocation: cathodal, anodal, or placebo. The three groups will be matched for age, gender, ethnicity, and audiometric main characteristics.

 It is a novel treatment of daily 20-min sessions for five consecutive days per week and for 2 consecutive weeks. It is very well described the inclusion-exclusion criteria, the selection of patients’ groups and the analysis of electrophysiological and subjective findings, which are done by statistical methods. Authors consider baseline evaluations, hearing assessment, primary and secondary outcomes. In difference to other studies, authors gave one-month follow-up period, in order to study the AEs of and tolerability of the patients

 2.           General comments

 Protocol design: the inclusion and exclusion criteria are well structured. Pre-treatment complete audiometric and neurologic examinations by experienced specialists was a good establishment.

 The balanced random allocation performed by cards using computer-generated random numbers, is a good idea, because it considers the age, gender, ethnicity, and audiometric main characteristics.

 As a part of a larger experiment, final results will be deterministic in intractable tinnitus treatment. The sample size may be greater than n=90, to improve the statistical significance

 3.           Constructive criticism

 It should be very interesting if authors show their data in corresponding plots to clearly visualize the patients’ distribution, electrophysiological and subjective readings and findings, pre- and post-treatment

 This study purposes the application of tDCS electric parameters. Authors use a specific device (by Mind Alive Inc.), it will be desirable if they show the precise electrical plot parameters (voltage, current, power and frequency) against time, performed with this device used.

I have read this submission. I believe that I have an appropriate level of expertise to confirm that it is of an acceptable scientific standard.

# Peer review: Referee response for version 1

**DOI:** 10.5256/f1000research.14726.r31942

**Published:** 2018-03-23

**Authors:** Leonid S. Godlevsky

**Affiliations:** 1 Ministry of Public Health, Odessa National Medical University, Odessa, Ukraine

Proposed protocol of investigation aims for the clinical effectiveness of tDCS in patients suffered from resistant to current therapy tinnitus assessment. Both cathode and anode role is in interest to verify based on the double blind method exploration.

 Hence, one month follow up along with filtering informative data from placebo effects and identification of adverse effects of tDCS are advantages of this protocol. Besides, two daily trials of tDCS during 5 days also points on the novelty of approach on the way of therapeutic potential of tDCS estimation in patients suffered from tinnitus.

 Such advantages of the protocol correspond to perfectly described methods, design, criteria on inclusion and exclusion, study protocol and outcomes assessments. Technology of tDCS is clear and reliable; all steps are controlled by researches. Statistical assessments are explicit for data gained in observations. Authors carefully consider all adverse effects which might encounter in tDCS delivering and must identify in their investigation.

 THI along with distress level will be used for the evaluation of the clinical effectiveness of tDCS. Meanwhile, background of the project contains data on quality of life (QL) worsening of patients with tinnitus. Despite QL is in good correlation with distress [1], it could be interesting to hear why just only distress was in scope and QL was not. QL was investigated by others cited authors just for estimation effectiveness of tinnitus treatment (N4 from list of literature- Khoramzadeh S.et al., 2016). The same question is for other disturbances mentioned in background chapter – such as sleep deteriorations, anxiety and depression development.

 It would be of worth to delineate criteria on resistance of patients suffered from tinnitus. Thus, it is interesting to hear details on their negative experience during previous drug treatment.

 Some notions on electrodes planned to use rose. In accordance to protocol the location of electrodes corresponds to 10-20 system of EEG electrode location. It is not enough for better imagine of the brain structures topography affected with your stimulations. Shape of electrodes is also important with such respect. Besides due to a flexibility of large electrodes the problem on non uniformity of currents under 3D electrodes leads to the increase of risks of burning of underlying tissues [2]. Hence, explanations on avoiding this problem are quite desirable. Also manufacturer of electrodes should be mentioned.

 It should to note that authors [3] did not use intensity of current higher than 1.5 mA when delivered stimulations through 35 cm ^2^ electrodes and emphasized on adverse effects of tDCS.  What is the reason for treating patients with higher intensity of electric current in your protocol? Parameters and regimes of planned interventions should have better ground. Namely, why you have justified 10 sessions? Is it settled for other brain pathology treatment?

 30 s period, which is planned to use for sham-stimulated patients might be distinguishable by them in the course of delivering 10 trials. Hence, proposed control via asking patients if they know what type of influence is going on looks rather reliable procedure for verifying blind type of investigation. But it should be mentioned if the moment of turning of stimulator was soundless or not, just for prevention patients training for the distinguishing between types of stimulation.

 General conclusion is that description of protocol on the tDCS treatment of patients suffered from tinnitus is rather convincing and deserves publication in highly ranked journal. Above mentioned minor remarks do not make serious impact upon very positive general estimation of proposed protocol of investigations. New and promising alternative for patients with tinnitus treatment is expected as result of its realization.

I have read this submission. I believe that I have an appropriate level of expertise to confirm that it is of an acceptable scientific standard.

## References

[1] HoffmannKKampMSteigerHJSabelMRappM: Correlation of psychooncological distress- screening and quality of life assessment in neurosurgical patients. *Oncotarget*.2017;8(67) : 10.18632/oncotarget.22802 111396-111404 10.18632/oncotarget.22802 29340062PMC5762330

[2] LenningerMFroeisTScheiderbauerMGrabherGBechtoldT: High current density 3D electrodes manufactured by technical embroidery. *Journal of Solid State Electrochemistry*.2013;17(8) : 10.1007/s10008-013-2108-1 2303-2309 10.1007/s10008-013-2108-1

[3] TuriZAmbrusGGHoKASenguptaTPaulusWAntalA: When size matters: large electrodes induce greater stimulation-related cutaneous discomfort than smaller electrodes at equivalent current density. *Brain Stimul*.7(3) : 10.1016/j.brs.2014.01.059 460-7 10.1016/j.brs.2014.01.059 24582373
